# Adverse neurologic events of immune checkpoint inhibitor monotherapy vs. combination therapy for melanoma

**DOI:** 10.1093/noajnl/vdaf030

**Published:** 2025-02-07

**Authors:** Nikita Das, Ravi Dhamija, David C Kaelber, Michael Kelly, Peter Xie, Deven Reddy

**Affiliations:** Case Western Reserve University School of Medicine, Cleveland, Ohio, USA; Case Western Reserve University School of Medicine, Cleveland, Ohio, USA; Department of Internal Medicine, Pediatrics, and Population and Quantitative Health Sciences, _Case Western Reserve University, Cleveland, Ohio, USA; Center for Clinical Informatics Research and Education, The MetroHealth System, Cleveland, Ohio, USA; Case Western Reserve University School of Medicine, Cleveland, Ohio, USA; Department of Neurological Surgery, The MetroHealth System, Cleveland, Ohio, USA; Department of Hematology/Oncology, The MetroHealth System, Cleveland, Ohio, USA; Case Western Reserve University School of Medicine, Cleveland, Ohio, USA; Department of Neurological Surgery, The MetroHealth System, Cleveland, Ohio, USA

**Keywords:** immune-related adverse events, immune checkpoint inhibitors, melanoma

## Abstract

**Background:**

Over the past decade, novel immune checkpoint inhibitors have revolutionized melanoma treatment. These new therapies are associated with complex immune-related adverse events. This study examines whether combination anti-PD-1/CTLA-4 immunotherapy for melanoma is associated with increased incidence of neurologic irAEs (n-irAEs) compared to anti-PD-1 monotherapy.

**Methods:**

A retrospective, multicenter study using TriNetX identified adult melanoma patients receiving anti-PD-1 monotherapy (pembrolizumab or nivolumab) (Cohort 1: *n* = 10,586) and patients receiving anti-PD-1/CTLA-4 combination therapy (nivolumab + ipilimumab) (Cohort 2: *n* = 5,705). Propensity score matching generated final cohorts (*n* = 5,185) using covariates: gender, race, age at diagnosis, TNM staging, nervous system metastasis, and history of neurologic disease. Odds ratios (OR) for n-irAE subtypes at 3- and 5-year post-therapy initiation were calculated, and Kaplan–Meier analyses assessed overall survival by aggregate n-irAE status in each cohort.

**Results:**

At 3 years, patients receiving combination immunotherapy exhibited increased risk of immune-related meningitis (OR: 2.6, 95% CI: [1.7, 4.1]) and encephalitis (OR: 3.0, 95% CI: [1.9, 4.9]), peripheral neuropathy (OR: 1.3, 95% CI [1.1, 1.5]), and myopathy (OR: 1.5, 95% CI: [1.1, 2.1]), but no significantly increased risk of demyelinating syndromes (OR: 1.5, 95% CI: [0.82, 2.6]), vasculitis (OR: 0.88, 95% CI: [0.43, 1.8]), or neuromuscular junction disorders (OR: 1.3, 95% CI: [0.87, 2.0]). At 5 years, these trends for risk of neurologic irAEs persisted. There was no significant difference in overall survival by n-irAE presence at 3 or 5 years in either cohort.

**Conclusions:**

Melanoma patients receiving combination anti-PD-1/CTLA-4 immunotherapy have greater long-term risk of n-irAEs than patients receiving anti-PD-1 monotherapy.

## Graphical Abstract

**Graphical Abstract:**
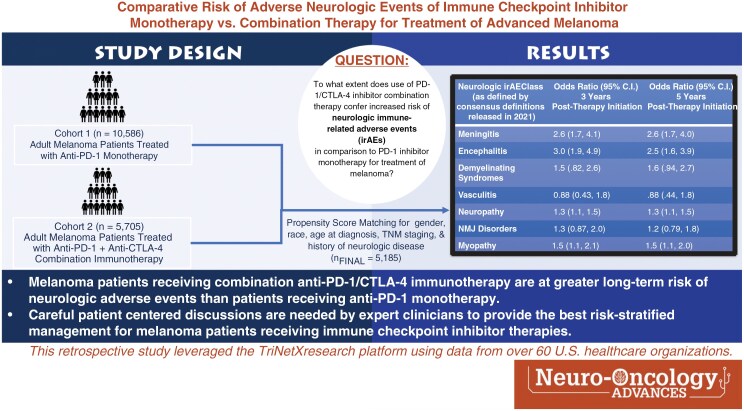

**Key Points**

- Combination ICI therapy for melanoma is associated with greater risk of neurologic irAEs than monotherapy that persists for 3–5 years after ICI initiation.
- Neurologic monitoring of melanoma patients on combination ICI therapy may optimize outcomes.

**Importance of the Study**

The landscape of advanced melanoma treatment has been profoundly shaped by the introduction of immune checkpoint inhibitors (ICIs), particularly PD-1 and CTLA-4 inhibitors, which have significantly improved patient outcomes. However, alongside their therapeutic benefits, use of these agents has brought forth immune-related adverse events (irAEs) that can impact various organ systems, including the nervous system. The recent publication of “Consensus disease definitions for neurologic immune-related adverse events of immune checkpoint inhibitors” provides a standardized framework for the evaluation and management of neurologic irAEs, underscoring the critical need for further research to align with these unified guidelines. Our study aims to address this by investigating, on a population level, the extent to which use of combination anti-PD-1/CTLA-4 immunotherapy is associated with a heightened long-term incidence of adverse neurologic events compared to anti-PD-1 monotherapy in melanoma patients. Understanding these risks highlights the need for vigilant neurological monitoring to improve patient care outcomes.

Melanoma, a malignancy of the pigment-producing cells of the skin, accounts for approximately 5% of all new cancer cases in the United States annually.^1^ Melanoma is a highly aggressive malignancy with historical 5-year survival rate of regionally advanced melanoma and metastatic melanoma estimated at 10% and 5%, respectively.^1,2^ The treatment of melanoma has improved significantly since the arrival of immune checkpoint inhibitor ipilimumab, an anti-CTLA-4 antibody, in 2011. The CheckMate-067 trial demonstrated improved median overall survival (mOS) and median progression free survival (mPFS) with combination therapy of nivolumab, a PD-1 inhibitor, with ipilimumab compared to monotherapy with ipilimumab.^3^ The 6.5-year outcome from CheckMate-067 was presented at ASCO 2021, reporting a mOS of 72.1 months with ipilimumab and nivolumab combination therapy compared to mOS of 36.9 months with nivolumab monotherapy and mOS of 8 months with ipilimumab monotherapy.^4^

Immune checkpoint inhibitors (ICI) targeting program cell death protein (PD-1) and cytotoxic T-lymphocyte-associated antigen 4 (CTLA-4) have demonstrated remarkable efficacy in treating advanced melanoma, leading to durable responses and improved survival rates.^5–7^ However, not all patients respond equally to these treatments, and some may experience immune-related adverse events (irAEs) due to inappropriate immune system activation. There is additionally increased risk of irAEs in patients treated with combination therapy of anti-PD-1 and anti-CTLA-4 compared to anti-PD-1 or anti-CTLA-4 monotherapies.^5^ IrAEs encompass a broad spectrum of side effects, such as skin reactions, gastrointestinal distress, endocrine dysfunction, liver inflammation, pneumonitis, nephritis, and colitis.^8^ These immunotherapies have also been tied to several neuropathologies.^9–11^ In 2021, consensus disease definitions for adverse neurologic irAEs (n-irAEs) of ICIs were released.^12^ The standardized agreement delineates meningitis, encephalitis, demyelinating disease, vasculitis, neuropathy, neuromuscular junction (NMJ) disorders, and myopathy as core subsets of autoimmune neurologic disorders related to ICI use.^12^

To date, there is a scarcity of literature reporting the incidence of neurologic irAEs, as defined by the Neuro irAE Disease Definition Panel, in melanoma patients. Though the cumulative incidence of neurologic side effects of anti-PD-1 and anti-CTLA-4 antibodies used to treat melanoma has been reported as less than 1% in results published from Phase III clinical trials, several Phase IV studies have estimated their incidence ranging up to 12%, with a median time to onset within 2 months of therapy initiation.^13–16^ Considering the increased mortality rate associated with neurologic adverse events in comparison to those of other organ systems—and also recognizing the impact of these complications on treatment strategies for patients—there is a need for a systematic characterization of the disease-specific neurologic irAE (n-irAE) risk profiles of ICIs according to current standards of care. The purpose of this study is to examine, at a population level, the extent to which use of anti-PD-1/CTLA-4 combination immunotherapy is associated with an increased incidence of adverse neurologic events in melanoma patients in comparison to monotherapy PD-1 inhibitor regimens.

## Materials and Methods

### Study Population

**Figure 1.**
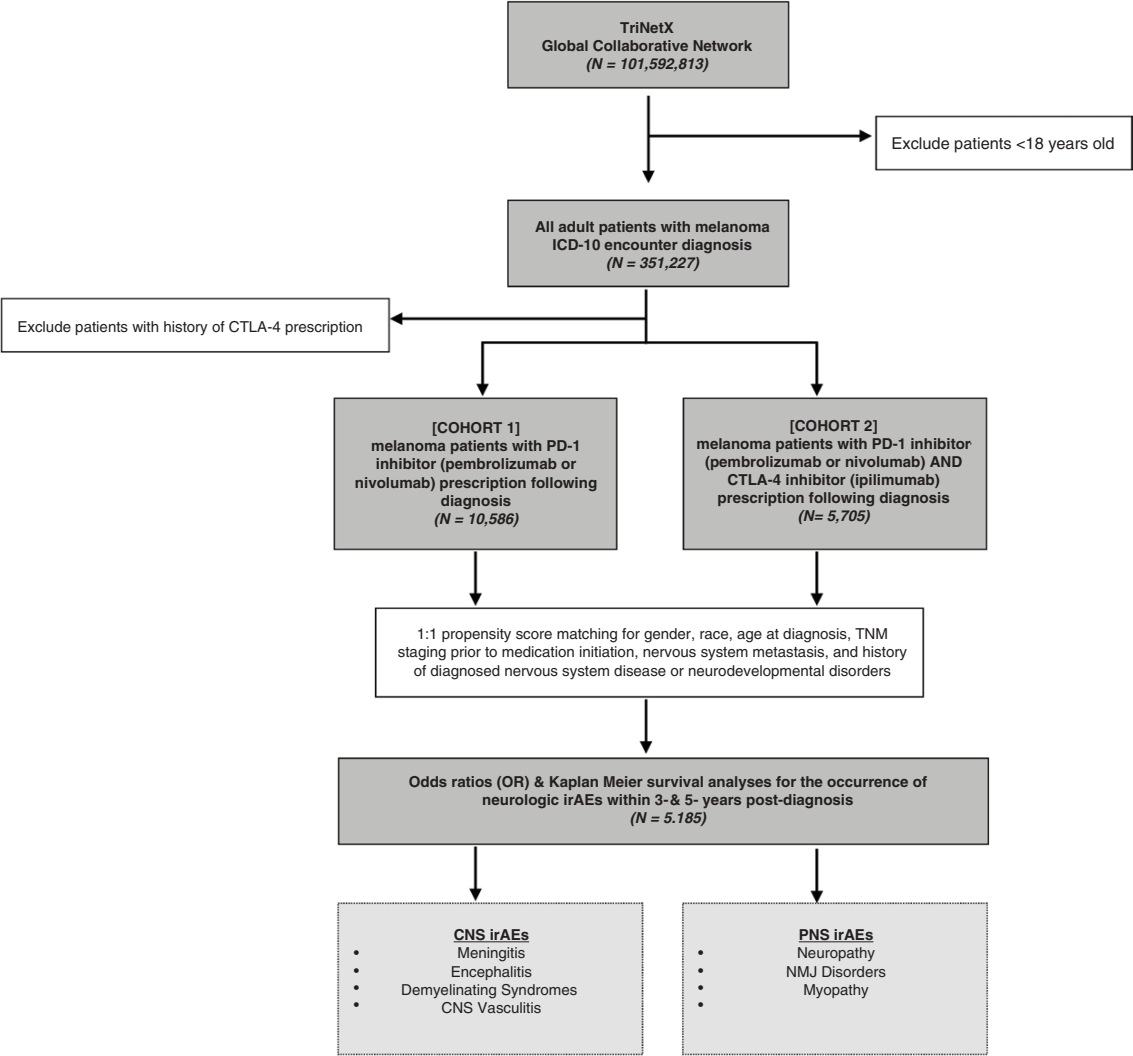
***Cohort Selection Methodology***—In accordance with STROBE guidelines, cohort selection within the TriNetX Analytics Platform was used to compare incidence of neurologic irAEs at 3 and 5 years following melanoma diagnosis and treatment with anti-PD-1 monotherapy (Cohort 1) or anti-PD-1 and anti-CTLA-4 combination therapy (Cohort 2).

A retrospective study was performed on the TriNetX platform—a health research network containing aggregated electronic health record (EHR) data from over 60 U.S. healthcare organizations—according to STROBE (Strengthening the Reporting of Observational Studies in Epidemiology) guidelines for cohort studies^17^ (Figure 1). TriNetX’s U.S. Collaborative Network was queried (January 2025) using International Classification of Diseases (ICD-10-CM) encounter diagnoses and RxNorm medication codes to identify adults diagnosed with melanoma and subsequently treated with PD-1 inhibitors (pembrolizumab or nivolumab) and CTLA-4 inhibitors (ipilimumab). Cohort 1 included patients with ≥1 melanoma ICD-10-CM encounter diagnoses with a PD-1 inhibitor prescription following diagnosis and no history of CTLA-4 inhibitor use following diagnosis. Cohort 2 included patients with ≥1 melanoma ICD-10-CM encounter diagnoses and ≥1 prescriptions for PD-1 and CTLA-4 inhibitors following diagnosis. Cohort 2 comprises patients who received combination immunotherapy at any point in time, irrespective of whether the treatments were initiated concurrently or sequentially. This study utilized de-identified data analyzed enmasse and was determined to be exempt from Western Institutional Review Board (IRB) approval as defined in Section §164.514(b)(1) of the HIPAA Privacy Rule.

### Statistical Design

To account for potential cofounders, propensity score matching (PSM) generated final cohorts using covariates for matching of gender, race, age at diagnosis, TNM staging prior to medication initiation, presence of brain, spine or other nervous system metastases (ICD-10-CM C79.3 & C79.4), and history of diagnosed nervous system disease or neurodevelopmental disorders. The primary outcome was new incidence of neurologic immune-related adverse event related ICD-10-CM encounter diagnoses, adapted from the “Consensus disease definitions for neurologic immune-related adverse events of immune checkpoint inhibitors” published in Journal for Immunotherapy of Cancer in 2021^12^ (Table 1). In attempt to isolate adverse events along the causal pathway of PD-1/CTLA-4 immunotherapy, patients with history of the corresponding irAE outcomes prior to melanoma diagnosis were excluded from their respective post-matching cohort analysis.

**Table 1.** Immune-Related Adverse Neurologic Events as defined by 2021 Neuro irAE Disease Definition Panel

Immune-related neurologic adverse event	Pathologies included in consensus definition	Corresponding ICD-10-CM codes queried
Meningitis	Aseptic Meningitis	A87, G03
Encephalitis	Encephalitis Meningoencephalitis Encephalomyelitis Limbic Encephalitis Cerebellitis Rhombencephalitis Opsoclonus-Myoclonus-Ataxia Syndrome Stiff-Person Syndrome/Progressive Encephalomyelitis	G04, G05, H55.89, G25.82
Demyelinating Syndromes	Optic Neuritis Transverse Myelitis Acute Demyelinating Encephalomyelitis Acute Hemorrhagic Encephalomyelitis Other Myelitis	H46, G36, G37, G04.82, G04.89, G04.91, G05.4, G04.00, G04.3
VASCULITIS	Primary Angiitis of the CNS Systemic Vasculitis with CNS Involvement	I67.7, I77.6
NEUROPATHY	Polyneuropathy Polyradiculopathy Axonal Polyradiculoneuopathy, radiculoplexus neuropathy CIDP AIDP Lewis-Sumner Syndrome MADSAM MMN GBS Variants (AMAN, AMSAN, MFS, cervical-brachial-pharyngeal variant) Sensory Neuronopathy Mononeuritis Multiplex, Vasculitis Neuropathy Brachial Neuritis, Lumbosacral radiculoplexus neuropathy Acute Mononeuropathy Cranial Neuropathy Small Fiber Neuropathy Autonomic Neuropathy	G60-G63, G65
Neuromuscular junction disorders	Myasthenia Gravis Lambert-Eaton Myasthenic Syndrome	G70, G73
Myopathy	Immune-mediated necrotizing myopathy Inflammatory Myopathy/Myositis	G72.4, G72.8, G72.9, M60

### Outcomes

Odds ratios (OR) were determined for the incidence of neurologic irAEs at 3- and 5-year post-therapy initiation. Kaplan–Meier analyses with Cox proportional hazards models were calculated to assess if presence of any n-irAE impacted survival probability within each cohort during the study period. To ensure patient outcomes were available for the entirety of the 3- or 5-year study period following therapy initiation, temporal censoring excluded patients from analysis who were prescribed the studied medications after January 2020. A *P*-value less than .05 was considered statistically significant.

## Results

**Figure 2.**
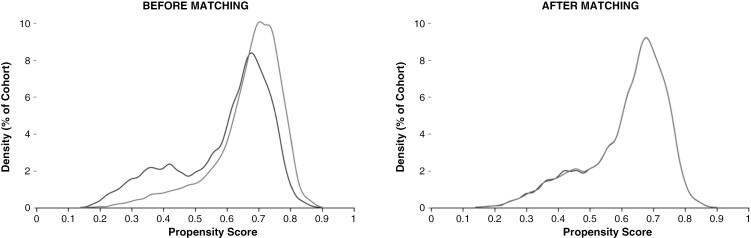
***Propensity Score Matching Results—*** Propensity Score Matching for covariates of gender, race, age at diagnosis, TNM staging prior to medication initiation, presence of brain or other nervous system metastasis, and history of diagnosed nervous system disease or neurodevelopmental disorders achieved a relatively symmetric distribution between cohorts. Cohort 1 (monotherapy) is represented in gray, while Cohort 2 (combination therapy) is represented in black.

Cohort 1 consisted of 10,586 patients with ≥1 ICD-10-CM encounter diagnoses for melanoma with subsequent PD-1 inhibitor prescriptions. Cohort 2 consisted of 5,705 patients with ≥1 prescriptions for anti-PD-1 *and* anti-CTLA-4 medications following an ICD-10-CM encounter diagnosis of melanoma. After matching, both cohorts included 5,185 patients (Table 2, Figure 2).

**Table 2.** Propensity Score Matching Results.

Table 2.1: Before Matching Demographics, Diagnoses, and Cancer Staging						
Covariate	Cohort 1 Mean ± SD	Cohort 2 Mean ± SD	# of Patients (% of Cohort 1)	# of Patients (% of Cohort 2)	Std. Difference	*P*-value
Age at Index	66.4 ± 14.6	61.6 + 14.2	10,586 (100%)	5,705 (100%)	0.345	< 0.0001*
Male	-	-	6,370 (60.2%)	3,460 (60.6%)	0.010	.570
Female	-	--	3,860 (36.5%)	2,050 (35.9%)	0.010	.574
Race—White	-	--	9,100 (85.9%)	5,045 (88.4%)	0.081	< 0.0001*
Race—African American	-	--	110 (1.04%)	65 (1.14%)	0.007	.705
Race—Asian	-	--	60 (0.57%)	46 (0.81%)	0.031	.068
Race—Unknown	-	--	1,025 (9.68%)	400 (7.01%)	0.095	< 0.0001*
Race—Other	-	--	291 (2.75%)	129 (2.26%)	0.026	.150
Prior Disease of Nervous System	-	--	5,200 (49.1%)	3,385 (59.3%)	0.212	< 0.0001*
Prior Neurodevelopmental Disorder	-	--	3,810 (36.0%)	2,330 (40.8%)	0.109	< 0.0001*
Metastasis to Brain/Meninges	-	--	880 (8.31%)	1,370 (24.0%)	0.440	< 0.0001*
Metastasis to Other Nervous System	-	--	210 (1.98%)	320 (5.61%)	0.198	< 0.0001*
Staging—Tumor Size (T)	-	--	1,590 (15.0%)	910 (15.9%)	0.028	.105
Staging—Nodal Infiltrate (N)	-	--	1,600 (15.1%)	905 (15.9%)	0.025	.148
Staging—Metastasis (M)	-	-	1,470 (13.9%)	775 (13.6%)	0.004	.861

Table 2.2: After Matching Demographics, Diagnoses, and Cancer Staging						
Covariate	Cohort 1 Mean ± SD	Cohort 2 Mean ± SD	# of Patients (% of Cohort 1)	# of Patients (% of Cohort 2)	Std. Difference	P-Value
Age at Index	61.9 ± 14.5	62.4 + 13.9	5,185 (100%)	5,185 (100%)	0.025	.263
Male	-	--	3,097 (59.7%)	3,127 (60.3%)	0.013	.532
Female	-	--	1,943 (37.5%)	1,890 (36.4%)	0.025	.227
Race—White	-	--	4,634 (89.4%)	4,570 (88.1%)	0.040	.046
Race—African American	-	--	68 (1.3%)	67 (1.3%)	0.003	.927
Race—Asian	-	--	31 (0.6%)	36 (0.7%)	0.008	.705
Race—Unknown	-	--	336 (6.5%)	381 (7.3%)	0.034	.098
Race—Other	-	--	116 (2.2%)	131 (2.5%)	0.023	.259
Prior Disease of Nervous System	-	--	2,888 (55.7%)	2,912 (56.2%)	0.011	.611
Prior Neurodevelopmental Disorder	-	--	2,038 (39.3%)	2,067 (39.9%)	0.012	.564
Metastasis to Brain/Meninges	-	--	833 (16.1%)	860 (16.6%)	0.015	.482
Metastasis to Other Nervous System	-	--	172 (3.3%)	189 (3.6%)	0.018	.375
Staging—Tumor Size (T)	-	--	704 (13.6%)	784 (15.1%)	0.044	.028*
Staging—Nodal Infiltrate (N)	-	--	707 (13.6%)	781 (15.1%)	0.042	.037*
Staging—Metastasis (M)	-	--	625 (12.1%)	684 (13.2%)	0.035	.082

Cohort 1: Melanoma patients with history of anti-PD-1 monotherapy, Cohort 2: Melanoma patients with history of anti-PD-1/CTLA-4 combination therapy following diagnosis.

### *3-Year Incidence of Neurologic Immune-Related Adverse Events* (Figure 3)

#### Central nervous system immune-related adverse events

0.53% of patients in Cohort 1 had an encounter diagnosis with meningitis within 3 years of their melanoma encounter diagnosis compared to 1.4% of patients in Cohort 2 (OR: 2.6 95% CI: [1.7, 4.1]). 0.43% of Cohort 1 patients had an encounter diagnosis with encephalitis within 3 years versus 1.3% Cohort 2 patients (OR: 3.0, 95% CI: [1.9, 4.9]). 0.39% of Cohort 1 patients had an encounter diagnosis for a demyelinating syndrome within 3 years of their encounter diagnosis of melanoma versus 0.57% in Cohort 2 (OR: 1.5, 95% CI: [0.82, 2.6]). 0.28% of Cohort 1 patients and 0.31% of Cohort 2 patients had an encounter diagnosis with vasculitis within 3 years of their melanoma encounter diagnosis (OR: 0.88, 95% CI: [0.43, 1.8]).

**Figure 3.**
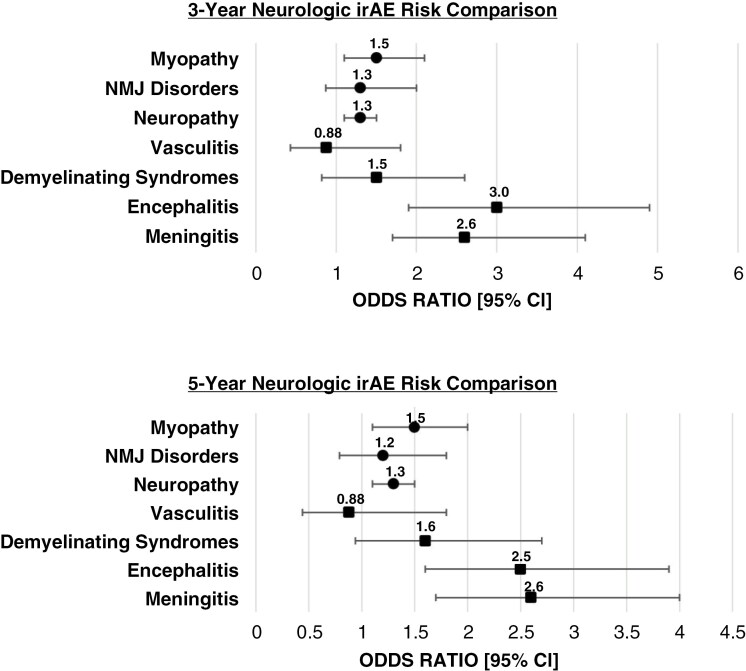
***Results Summary—*** This forest plot represents the Odds Ratios calculated for incidence of neurologic irAEs in melanoma Patients treated with anti-PD-1/CTLA-4 combination therapy (Cohort 2) in comparison to patients treated with anti-PD-1 monotherapy at 3 and 5 years following therapy initiation [Circles: Peripheral Nervous System irAEs, Squares: Central Nervous System irAEs].

#### Peripheral nervous system immune-related adverse events

6.4% of Cohort 1 patients had an encounter diagnosis of neuropathy, compared to 8.0% of Cohort 2 patients within 3 years of immunotherapy initiation (OR: 1.3, 95% CI [1.1, 1.5]). 0.73% of Cohort 1 had an encounter diagnosis of a NMJ disorder within 3 years versus 0.97% of patients in Cohort 2 (OR: 1.3, 95% CI: [0.87, 2.0]). 1.5% of Cohort 1 patients had an encounter diagnosis of myopathy, compared to 2.3% of Cohort 2 (OR: 1.5, 95% CI: [1.1, 2.1]).

### 5-Year Incidence of Neurologic Immune-Related Adverse Events (Figure 3)

#### Central nervous system immune-related adverse events

At 5 years following therapy initiation, patients receiving anti-PD-1/CTLA-4 combination immunotherapy exhibited significantly higher risk for immune-related meningitis (1.4% vs. 0.56%, OR: 2.6, 95% CI: [1.7,4.0]) and encephalitis (1.3% vs. 0.52%, OR: 2.5, 95% CI: [1.6, 3.9]) encounter diagnoses. The risk of ≥1 encounter diagnoses for vasculitis as a treatment-emergent complication was equivalent between cohorts at 0.3% (OR: 0.88, 95% CI: [0.44, 1.8], and no significant difference was identified between cohorts with respect to demyelinating syndromes (0.68% vs. 0.43%, OR: 1.6, 95% CI: [0.94, 2.7]) (Figure 3).

#### Peripheral nervous system immune-related adverse events

At 5 years following therapy initiation, Cohort 2 exhibited significantly higher risk of neuropathy (8.7% vs. 6.8%, OR: 1.3, 95% CI: [1.1, 1.5]) and myopathy (2.3% vs. 1.5%, OR: 1.5, 95% CI: [1.1, 2.0]) encounter diagnoses. An increased but insignificant risk for NMJ disorder encounter diagnoses was observed in Cohort 2 (1.0% vs. 0.9%, OR: 1.2, 95% CI: [0.79, 1.8]).

### Survival Trends with Respect to Neurologic irAEs

At 3 years following therapy initiation, post-PSM Kaplan–Meier analyses revealed no significantly increased risk of death in patients with any neurologic irAE after receiving anti-PD-1 monotherapy (Cohort 1) in comparison to patients with no history of n-irAEs after anti-PD-1 monotherapy initiation (hazard ratio [HR]: 1.15, 95% C.I. [1.0, 1.3]). At 5 years following therapy initiation, there was no significant difference in overall survival for Cohort 1 patients with n-irAEs compared to those with no documented n-irAE (HR: 1.13, 95% C.I. [1.0, 1.3]). For patients with history of combination immunotherapy (Cohort 2), there was no significant difference in overall survival of patients who experienced any n-irAE versus those who had no history of n-irAEs at 3 years [HR: 1.02, 95% C.I. [0.91, 1.2]) or 5 years following therapy initiation (HR: 1.04, 95% C.I. [0.92, 1.2]).

## Discussion

In the past decade, the U.S. FDA approval of PD-1 and CTLA-4 inhibitors has revolutionized treatment of advanced melanoma. Before their introduction, treatment options included surgery, chemotherapy, targeted therapies (BRAF and MEK inhibitors), and immunotherapies such as interleukin-2 and interferon-alpha.^18,19^ These treatments had limited efficacy and significant side effects.^20^ Several pivotal trials have since demonstrated the superiority of PD-1 inhibitors compared to traditional therapies for melanoma. Pembrolizumab and nivolumab have been consistently demonstrated to have better response rates, progression-free survival, and overall survival compared to chemotherapy in advanced melanoma patients.^7,21^ Combination therapy of nivolumab and ipilimumab has shown survival advantage over nivolumab and ipilimumab monotherapy.^4^ Novel immune checkpoint inhibitors are now the standard of care first-line therapy for metastatic melanoma for eligible patients. With the advancement and widespread use of immunotherapy, we now face new treatment-related side effects and challenges in immune-related adverse events.^22^

This study assesses trends in incidence of neurologic irAEs in melanoma patients undergoing PD-1 inhibitor monotherapy compared to those receiving both anti-PD-1 and anti-CTLA-4 immunotherapies over time, unveiling insight into the relative risks associated with these treatment regimens. While the incidence of nervous system side effects of these immune checkpoint inhibitors has previously been estimated between 1% and 12%,^8–10,13,22^ this investigation using cohorts of more than 5,000 melanoma patients found that the incidence of the various subtypes of neurologic irAEs ranges between 1% and 9%. The risk of neurologic autoimmune complications is small yet may have important implications for treatment adherence and course. A recent meta-analysis performed by Farooq et al. uncovered an increased incidence of cumulative neurologic irAEs following ICI treatment in comparison to chemotherapy amongst patients with various types of malignancies.^16^ While the association between ICI treatment and neurologic irAEs has been convincingly established, existing reports on this topic have largely relied on published data from clinical trials, whose generalizability may be limited by stringent inclusion and exclusion criteria. Our present study juxtaposes existing explorations by highlighting prospectively collected patient experiences, thereby providing a more complete, disease-specific clinical picture of the immuno-oncological phenomenon at hand. Not only have we uncovered the specific neurologic risk profile of ICIs amongst the melanoma patient population according to recently standardized definitions, but also, we provide a timeline over which to evaluate the magnitude of these risks over time.

### Hierarchical Prevalence of Neurologic irAEs

Our findings reveal significantly different risk profiles for immune-related neurologic complications in melanoma patients treated with PD-1 inhibitor monotherapy versus those treated with both PD-1 and CTLA-4 inhibitors. Of the studied neurologic irAEs, peripheral neuropathy was the most prevalent condition followed by myopathy, meningitis, encephalitis, NMJ disorders, demyelinating syndromes, and vasculitis. The differences in incidence likely stem from varying susceptibilities of different neurological structures to immune-mediated attacks.^6,23^ Peripheral neuropathy may be more common of the neurologic irAEs due to their increased baseline exposure to immune cells compared to the central nervous system (CNS).^24^ Meanwhile, conditions like encephalitis may occur less frequently due to the blood-brain barrier providing some protection against immune-mediated reactions.^25^

Incidence of CNS irAEs like meningitis and encephalitis is significantly higher in melanoma patients with history of anti-PD-1/CTLA-4 combination therapy than in those treated with anti-PD-1 monotherapy. This trend is paralleled by peripheral nervous system (PNS) irAEs neuropathy and myopathy, which demonstrated over one-fold higher risk of these irAEs in patients receiving combination therapy. Our data suggest that patients using both anti-PD-1 and anti-CTLA-4 therapies have a higher risk of CNS irAEs than PNS irAEs. The difference in magnitude of odds ratios for these irAE subclasses may stem from several factors including differential expression patterns of immune checkpoint molecules between these systems or even diagnostic challenges related to symptom severity and presentation of CNS irAEs in comparison to PNS irAEs.^6,26^ In the CNS, immune privilege is a defining feature, characterized by restricted local immune responses. This is partly due to the high expression of Fas ligand (FasL) and the limited constitutive expression of major histocompatibility complex (MHC) class II antigens, which are typically only induced after injury. This immune-privileged environment facilitates the elimination of infiltrating lymphocytes through apoptosis, a phenomenon commonly observed in injured CNS tissues.^27^ In contrast, the PNS lacks the same level of immune privilege. MHC class II antigens are constitutively expressed in the PNS, enabling a more robust immune response. Moreover, injured PNS tissues accumulate significantly greater numbers of endogenous T cells compared to the CNS, and these T cells are less prone to undergoing apoptosis.^26,27^ These differences in immune dynamics between the CNS and PNS likely contribute to the variation in the presentation and detection of irAEs within these systems, potentially influencing the observed disparities in odds ratios. Interestingly, though there was a slightly increased incidence of vasculitis and NMJ disorder complications in melanoma patients receiving both PD-1 and CTLA-4 inhibitor therapies, this difference was not statistically significant. It is conceivable that the addition of CTLA-4 inhibitors does not compound the risk for these specific irAEs because of extraneous features of the cardiovascular system and the NMJ which may protect these microenvironments from autoimmune destruction to a greater extent than structures affected by the other neurologic irAEs.^6,24–27^

### Persistence of Neurologic irAE Risk over Time

From various immunotherapy clinical trials thus far, it has been established that upfront irAE risk is higher with combination PD-1/CTLA-4 inhibitors than PD-1 inhibitor therapy alone, because CTLA-4 provides less specific checkpoint inhibition than PD-1 targeted therapy.^13^ However, some long-term data seem to suggest that the irAE risk may become comparable over time.^6,13^ When comparing the incidence of neurologic irAEs at 3 years following therapy initiation to 5 years, the increased risk of neurologic irAEs in melanoma patients receiving PD-1/CTLA-4 combination therapy persists over time, at a similar magnitude for most of the neurologic irAEs except encephalitis and NMJ disorders whose odds ratios were marginally decreased at the 5-year mark (Table 3). This trend indicates a relatively stable baseline risk for each of the neurologic irAE subclasses. The data show that a patient’s risk of neurologic irAEs remains relatively consistent over time, with patients receiving combination ICI therapy at greater risk than those receiving monotherapy.

### Neurologic irAEs Are Not Associated with Increased Mortality Risk

While this investigation primarily aimed to examine the incidence of various classes of n-irAEs in melanoma patients undergoing monotherapy or combination immunotherapy, our secondary intra-cohort analyses provided valuable insights into the relationship between n-irAEs and overall survival in this population. Notably, the prevalence of any neurologic irAE did not significantly impact overall survival rates at 3 or 5 years following therapy initiation, regardless of treatment modality. This finding contrasts with a recent study by Pepys et al. in 2023 which reported that n-irAEs were associated with improved survival (HR = 0.4, 95% C.I. (0.32–0.77) in 937 patients with advanced melanoma.^28^ While our present study does not reflect this trend, the discrepancy may be explained by the hypothesis that n-irAEs signify a robust immune response, which may be beneficial for tumor control without compromising survival. This perspective is bolstered by other recent studies demonstrating that advanced melanoma patients developing any kind of irAE often experience improved survival outcomes compared to those who do not develop irAEs.^29–31^ Additionally, irAE management strategies, such as the use of systemic corticosteroids and other immunosuppressive agents, have no adverse impact on overall survival or time to treatment failure in melanoma patients treated with ipilimumab.^32^ Collectively, these findings highlight that n-irAEs, when effectively managed, may play a neutral or even favorable role in shaping patient outcomes. They further emphasize the importance of timely intervention to mitigate the potential adverse effects of n-irAEs while preserving the long-term benefits of immunotherapy.

### Relevance and Future Directions

The findings of this study have significant implications for the course of cancer care. Careful patient centered discussions are needed by expert clinicians to provide the best risk-stratified management for patients. Guidelines from the Society for Immunotherapy of Cancer recommend prompt evaluation and intervention upon suspicion of immune-related neurological adverse events.^33^ Achieving this requires multidisciplinary approaches involving neurologists, oncologists, and internal medicine practitioners to ensure early recognition and appropriate management. The present investigation clarifies the importance of vigilant monitoring for neurological symptoms in patients undergoing immunotherapy. This includes educating patients about symptoms associated with these neurologic conditions and conducting regular neurological assessments during outpatient treatment. Given that most irAEs are diagnoses of exclusion, understanding prevalence allows for earlier consideration of these conditions in differential diagnoses, potentially avoiding unnecessary delays by prioritizing appropriate imaging and lab studies to rule out mimics. There is a need for development of standardized screening tools for irAEs. Future research may examine the individual neurologic irAE profiles of each ICI medication separately or further discerning the average time frame for emergence of each neurologic irAE subclass.

Overall, this study provides evidence that combination ICI immunotherapy is associated with a long-term increased risk of neurologic irAEs meningitis, encephalitis, neuropathy and myopathy in melanoma patients. It is worth noting that factors like tumor mutation burden, heterogeneity of PD-1 and CTLA-4 expression in host and malignant cells, and other patient-specific characteristics may influence a patient’s risk of experiencing neurologic irAEs. It may be important to consider if patients have prior history or family history of any immune mediated neurological complications before deciding treatment regimen, since this could be an indicator for their propensity to develop irAEs after beginning anti-PD-1/ CTLA-4 treatment.^34,35^ As we enter the age of precision medicine, identifying biomarkers that predict the onset of immune-related adverse events will be essential for enhancing efficacy and minimizing side effects of ICIs.

### Limitations

This study has inherent limitations characteristic of retrospective cohort analyses. Relying on existing electronic health records (EHR) introduces potential challenges, including misclassification, incomplete data, and confounding factors that may affect the accuracy of our findings. Additionally, retrospective studies cannot establish causality, limiting the specificity of the conclusions. Our use of the TriNetX multicenter, deidentified database allowed for a large-scale analysis of real-world data, offering insights that would be difficult to achieve through single- or multi-institution studies. However, this approach also presented specific limitations. We were unable to verify that all neurologic irAE cases were entirely free of infectious contributors, as TriNetX does not report specific laboratory values, such as cerebrospinal fluid culture results, which are critical for confirming diagnoses. Additionally, while efforts were made to exclude infectious causes using diagnostic codes, the limitations of EMR coding and the inability to perform individual chart reviews meant that complete exclusion of infectious contributors could not be ensured. Unlike a single-institution retrospective cohort study, where data pulls can be manually audited, this study relied on aggregated data, which restricts the ability to validate individual patient characteristics. Furthermore, differences in baseline disease severity between the cohorts could have influenced the observed outcomes. Patients receiving combination immunotherapy may have had more advanced melanoma or worse prognoses, despite our adjustments for factors like nervous system metastases and TNM staging. These residual disparities likely impacted survival analyses, particularly given the incomplete availability of TNM data across the cohort. Despite these limitations, the study provides the largest and most comprehensive risk analysis to date of neurologic irAEs in melanoma patients treated with combination anti-PD-1/CTLA-4 immunotherapy versus anti-PD-1 monotherapy. The pragmatic design highlights real-world trends in a diverse patient population, offering valuable insights that complement findings from more controlled, smaller-scale studies.

## Conclusion

This study provides evidence that melanoma patients receiving PD-1 inhibitor therapy in combination with CTLA-4 inhibitor therapy are at greater risk of experiencing immune-related neurologic adverse events than patients on anti-PD-1 monotherapy treatment regimens. Importantly, the presence of neurologic irAEs was not observed to impact 3- and 5-year survival probabilities, irrespective of treatment modality. These findings underscore the importance of vigilant monitoring and management of neurologic irAEs in melanoma patients undergoing immune checkpoint inhibitor immunotherapy. Further research is warranted to refine predictive markers for irAEs and develop tumor-specific immune checkpoint inhibitors to minimize risks of irAEs while optimizing the therapeutic benefits of ICI immunotherapy for the treatment of advanced melanoma.

## Data Availability

The data that support the findings of this study are available, pending institutional approval, upon reasonable request from the corresponding author [D.R.].

## Previous Presentations

American Association of Neurological Surgeons 2024 Annual Meeting

Oral Presentation, May 5, 2024, Chicago, IL, USA *(Preliminary Results)*

29^th^ Annual Meeting and Education Day of the Society for Neuro-Oncology (SNO)

Published Abstract & Presentation, November 21-24, 2024, Houston, TX, USA *(Final Results)*
